# Vaccines and Antiviral Drugs in Pandemic Preparedness

**DOI:** 10.3201/eid1201.051068

**Published:** 2006-01

**Authors:** Arnold S. Monto

**Affiliations:** * University of Michigan School of Public Health, Ann Arbor, Michigan, USA

**Keywords:** Influenza, pandemic viruses, antiviral drugs, vaccines, perspective

## Abstract

While measures such as closing schools and social distancing may slow the effects of pandemic influenza, only vaccines and antiviral drugs are clearly efficacious in preventing infection or treating illness. Unless the pandemic strain closely resembles one already recognized, vaccine will not be available early. However, studies can be conducted beforehand to address questions concerning vaccine dose, frequency of inoculation, and need for adjuvants. In contrast, antiviral drugs, particularly the neuraminidase inhibitors, will be effective for treatment and available if stockpiling takes place. Special questions need to be answered if a highly lethal virus, such as influenza A (H5N1), produces the pandemic. Both vaccines and antiviral drugs will be required for a coordinated strategy.

## Abstract 2

Controlling a pandemic with vaccine and antiviral drugs will require a coordinated international approach to determine how the least amount of virus can immunize the largest segment of a population.

Prevention of influenza, particularly during a pandemic, may be attempted by many measures, such as closing schools, using facemasks, and keeping infected persons away from those susceptible, now termed social distancing. However, none of these measures are of clear value in preventing infection, even if they could be accomplished. A principal reason little effort has been made to determine their usefulness in the interpandemic period is the usual availability of vaccine, which is of known value in prevention. Thus, few studies have been undertaken. Similarly, symptomatic therapy is possible and perhaps appropriate in treating milder illnesses. Antimicrobial drugs are necessary when bacterial complications occur. However, antiviral drugs are specific and can not only prevent infection but also treat illness (*1*).

A pandemic virus will likely spread so rapidly from the source that vaccine availability may be delayed for months after major outbreaks begin. In addition, much of the population will be totally susceptible. We will likely not be able to prepare stockpiles of virus concentrates well matched with the pandemic strain for vaccine production before the strain has actually shown itself. In contrast, antiviral drugs, particularly the neuraminidase inhibitors (NAIs), will be effective against any pandemic virus, and stockpiling is possible (*1*). However, supplies will likely be limited, even with a relatively large stockpile, and may well be exhausted without careful planning before vaccine is available.

## Vaccines: Needs and Priorities in the Prepandemic Phase

Key to the ability to have vaccines ready is early detection of the pandemic virus. Improved surveillance networks are vital for this purpose. While the specific variant that emerges will probably be different antigenically from any recognized, much can be learned by studying the known variants of likely subtypes. An example of what needs to be done before the pandemic is the concerted evaluation in 1976 of a virus variant thought to have pandemic potential (*2*). The swine influenza virus, detected in humans in that year, was viewed as a pandemic threat. Because the pandemic never occurred, researchers had time to complete a large range of pediatric and adult studies. We learned that those who had no previous experience with that subtype needed to be vaccinated twice with a split preparation. The whole-virus vaccine then commonly used could not be given to those persons without frequent systemic reactions, but the whole-virus vaccine was more immunogenic and might be acceptable if rapid response was desired. In persons previously exposed to the influenza virus subtype, the whole-virus vaccine was much less reactogenic and appeared more immunogenic than the split product (*3**,**4*). These observations still have relevance in the current situation.

Similar studies need to be carried out now on all subtypes of pandemic potential. However, we cannot do so without choosing priorities, given restrictions of time and resources. Choices must be made on the basis of historic and current observations. At one time, a closed, fixed cycle of type A subtypes was thought to exist, with one following the other, each producing a pandemic (*5*). This theory predated molecular analysis of the hemagglutinin of the viruses and was based as a classification system derived from their epidemiologic characteristics. As shown in the Table, the concern that swine influenza would appear in 1976 was supported by seroarcheology, evidence in serum samples collected before, for example, 1968 that an A (H2) and A (H3) virus had previously circulated (*6*). Few currently believe this theory in its entirety, since it would require that a subtype remain undetected in a host, perhaps in humans, for a long period of time. However, the determination, using molecular techniques unavailable until well after the pandemics had occurred, that the A (H2N2) and A (H3N2) viruses were reassortants between previous human and avian strains suggested a different origin for these viruses (*7**,**8*). The avian predecessors of these 2 new viruses were not highly pathogenic, and the resultant pandemics showed a typical U-shaped death rate, highest in the very young and old. The 1918 virus had a different derivation and was apparently not a reassortant but a mutant. It also had an avian origin, but the progenitor virus has not yet been identified, so its pathogenicity in birds is unknown (*9*). However, its epidemiologic signature in humans was high case fatality in young adults (*10*).

**Table:** Influenza A subtypes in humans

Year of recognition	Old terminology	Molecular antigenic terminology
1889		H2*
1902		H3*
1918	Swine influenza	H1N1
1932	A0	H1N1
1947	A prime	H1N1
1957	Asian	H2N2
1968	Hong Kong	H3N2
1976	Swine	H1N1†
1977	Russian	H1N1

*Unknown N subtype. †Limited human-to-human transmission.

The question, then, based on this evidence, is which viruses should be studied to prepare a vaccine to control the next pandemic? Will type A (H2N2) return, in keeping with the recycling theory? Much of the population will now be susceptible. Type A (H9N2), a less pathogenic avian virus, has transmitted occasionally to humans, with little or no further transmission, but has not produced disease with high case fatality (*11*). The highly pathogenic type A (H5N1) virus is at the top of the list of potential pandemic threats. This virus, if it becomes adapted for human transmission without a reduction in virulence, could result in a pandemic far worse than 1918, also involving healthy, younger persons (*12**,**13*). Other viruses, such as the A (H7) highly pathogenic avian strains, including A (H7N7), which infected humans in the Netherlands, and A (H7N3), which spread extensively in western Canada, can also be considered candidates but are not as high on the list since fewer transmissions to humans and less clinical disease have been seen (*14**,**15*).

## Prepandemic Vaccine Evaluation

Scientific questions that have been raised concerning the various priority potential pandemic viruses are different, depending on the specific subtypes. The goal in all cases is production of an immune response with the least amount of antigen, so that more doses can be available. Perhaps the simplest situation is that of A (H2N2), a known quantity, because of its presence from 1957 to 1968, in terms of immune response, population likely to be infected, and expected disease characteristics. Also, that virus presents the fewest issues about vaccine production, for the same reasons. However, the basic question relates to producing the best immune response with the least amount of antigen and avoiding if possible the need for a second injection, which would use additional antigen and delay production of protective immunity. One approach, already studied, is to leave the harvested virus particles intact, the modern equivalent of the whole-virus vaccines evaluated in 1976 (*16*). In persons without prior infection with this virus, 1 injection of as little as 3.8 μg with alum, a widely used adjuvant, produced some antibody response, as determined by the hemagglutination-inhibition (HAI) test, traditionally used to assess protection afforded by inactivated vaccines. A second injection produced high titers. Positive features of this approach are that vaccine could be produced more quickly, and antigen would be spared. A possible negative feature would be reactogenicity in children. However, we do not know whether, with modern purification methods, these vaccines would have the reactogenicity of those produced in 1976. Less work has been done with avian influenza, A (H9N2), but similar approaches might be used with these nonhighly pathogenic avian viruses (*16**,**17*).

The highly pathogenic A (H5N1) virus presents many more problems in vaccine development and evaluation. The first one, already solved, involves removal of the molecular motif of high pathogenicity, the multibasic cleavage site, from the hemagglutinin. The virus is then reassembled by using reverse genetics, but on a background of the high-growth type A virus, PR8 (*18*). Producing vaccine by using this engineered virus can then proceed without high-level containment. However, we know from previous work with a less pathogenic influenza, A (H5N3) that antibody response to this avian subtype is not good and that adjuvants and multiple doses are required (*19**,**20*). The A (H5N3) vaccine was given to only small numbers of healthy adults. Response did not occur in persons given <30 μg of antigen alone but did in persons given the antigen with the MF-59 adjuvant. However, after 16 months, essentially no antibody was seen even in those who received the vaccine with adjuvant. On revaccination with the same preparation, persons previously given the vaccine with adjuvant had an anamnestic response, while persons given the unadjuvanted vaccine again had a poor response. Measuring and evaluating the meaning of the antibody response to some avian viruses is also an issue. Even infection with the A (H5N1) virus does not produce a good HAI antibody response; the antibody needs to be detected with a neutralization test (*21*). Similarly, neutralization testing is necessary to detect response to vaccine; however, a specific level of HAI antibody has been associated with protection, but no similar correlate of neutralization antibody has yet been developed (*5*).

Further evaluation of dosage and need for booster injection of these vaccines is in process. An international agenda is needed so that the diverse issues will be systematically investigated. Several high-priority vaccines need to be evaluated at various frequencies of administration and dose levels, with and without adjuvants. No single country can do it adequately (*10*). The work has started, especially with the A (H5N1) vaccine produced by reverse genetics, but the research has a long way to go.

## Antiviral Drugs: What Can be Done Before the Pandemic

With antiviral drugs, the scientific questions that need to be answered before the pandemic are not as daunting (*13*). Originally, both classes of antiviral drugs were believed to be effective against a pandemic virus. Adamantane action is limited to type A viruses, but all pandemic viruses are type A (*15*). The neuraminidases of many different type A viruses have been evaluated with respect to NAIs, and all have been found susceptible (*1*). As a result, given advance planning so that supplies are available, antiviral drugs can be used early in a pandemic and do not require specific production and formulation. Because they are much less costly than NAIs, adamantanes were part of the overall antiviral strategy (*20*). Having 2 classes of drug increased the amount of antiviral drugs available to stockpile since production limitations are an issue with the NAIs.

Considerable evidence indicates that both classes of drugs work well in prophylaxis against susceptible seasonal influenza viruses and that prophylaxis does not increase resistance. In fact, amantadine prophylaxis has been tested in a pandemic situation, and while efficacy may be reduced in persons with no previous exposure, which seems to increase protection, it is still 70%–80% (*22*). Although no direct comparisons have been carried out with the adamantanes, NAIs appear at least as efficacious. The 2 NAIs, zanamivir and oseltamivir, gave similar results when given daily for 4 or 6 weeks (*23**,**24*). They may be more efficacious in preventing febrile illnesses, although asymptomatic infection often still occurs. This characteristic is actually desirable, since it provides protection against the next wave of the pandemic virus. However, in some cases, infection is prevented completely, so vaccine should be used when available.

In treatment, adamantanes and NAIs diverge in their efficacy. No reliable data on use in pandemics exists, and no head-to-head studies have been carried out. Studies of treatment with amantadine and rimantadine did not allow firm estimates of how much they shortened duration of illness but were sufficient to conclude that they produced more rapid resolution than symptomatic therapy, such as aspirin (*25*). No data suggest that they prevented complications in any population; indeed, recent experimental studies suggest that they do not (*26*). However, the main reason they have never been considered for therapy in a pandemic is that antiviral resistance occurs in >30% of those given the drug for treatment and that resistant viruses are fully pathogenic and transmissible (*27*). While resistance occurs when oseltamivir is used in treatment, it is far less frequent than with the adamantanes, and the mutant viruses may be less infectious and transmissible than wild type (*28**–**30*). This conclusion cannot be viewed as absolute; with high-volume use, which has occurred thus far only in Japan, resistant viruses could begin to circulate. Emergence of resistance has apparently occurred with adamantanes, and the more recent type A (H5N1) virus, as well as some currently circulating seasonal viruses, are not susceptible to this drug class.

Another advantage of NAIs in therapy is their ability to prevent certain complications (*31**,**32*). Some evidence also shows increased efficacy in illnesses that are identified as more severe at onset (*33*). We cannot predict how this efficacy would translate into treatment success in a pandemic, but it encourages using them to treat persons who are recognized early to be more symptomatic.

With ordinary influenza viruses of pandemic potential, such as type A (H2N2) and A (H9N2), treatment success in the interpandemic period would be more likely relevant to the pandemic. Such may not be the case with the type A (H5N1) virus. The virus has evolved since the 1997 Hong Kong outbreak, and some evidence of a systemic infection involving the brain and gastrointestinal tract exists (*12**,**34*). This infection has also been demonstrated in laboratory animals such as ferrets (*35*) and means that the drug may need to reach adequate concentration in these sites, remote from the respiratory tract. Zanamivir is not orally bioavailable and is thus not likely to be useful in treating influenza A (H5N1) infection, although it might play a role in prophylaxis. Oseltamivir, in contrast, is absorbed and metabolized. While human studies of oseltamivir in treatment would be critical now, such studies have been difficult to carry out, since the disease has been occurring in areas where recognition of the cause is often delayed. We have yet to determine whether the mixed results that have been described with this drug in the limited case reports are due to late treatment or other factors, such as need for higher doses (*13**,**35*). A planned clinical trials network may solve this problem. In the meantime, animal studies are urgently needed to evaluate dosage and duration of therapy, particularly against the Vietnam strain of the A (H5N1) virus. These studies would help guide treatment of human cases until more data are available. Mouse studies have already indicated that, while oseltamivir is effective, it is not as effective when given for 5 days as it was against the 1997 Hong Kong variant of A (H5N1) influenza (*36*). This finding indicates that treatment for 10 days might be necessary, since in the mouse studies, replication resumed after therapy was stopped. The dose may also need to be increased. Studies in ferrets and nonhuman primates would have more relevance to the situation in humans than studies in mice.

## Vaccine Activities in the Pandemic

Countries will need to have pandemic plans in place to establish priorities for vaccine use. However, to help refine these decisions once the pandemic begins, epidemiologic- and vaccine-related issues will have to be addressed. The pandemic must be characterized not only in terms of the groups infected but also, more importantly, case fatality in each group. Vaccine supply will be increasing over time, so the question is which groups should get it earlier. Current pandemic planning usually directs vaccine to the groups who traditionally have had the highest death rates, mainly the old and the very young, but this might have to change. If the 1918 pattern repeated itself, or for example, if the A (H5N1) virus produces the pandemic and does not change in virulence or its tendency to infect the young, vaccination priorities would have to be changed radically.

Once the pandemic virus is available, a rapid evaluation will be needed to address questions of dosage, need for adjuvants, and booster vaccination. However, this evaluation will need to be done quickly, especially for regions of the world close to the pandemic origin, so as much work as possible should be done before the pandemic. First, though, a virus for vaccine production will need to be created from the pandemic strain, with appropriate manipulation to make it high yielding. In the process, the molecular and antigenic differences between this virus and those of the same subtype already available will need to be defined. With luck, the pandemic virus may be similar enough to one already studied so that any available concentrates can be used. However, similarity is unlikely because of the antigenic variation of influenza strains within a subtype. Rather than stockpiling, another strategy needs to be considered for vaccines containing a virus such as A (H5N1) for which vaccine development has already begun. That virus can be included in vaccines in use before the pandemic. Although influenza A (H5N1) virus has been evolving, even a poorly matched vaccine might provide some protection, especially against a variant with such high lethality (*37*). Also, if 2 injections of a specific vaccine are necessary, an older vaccine could prime, so that only 1 injection of the new vaccine would be needed. An A (H5N1) vaccine might initially be directed for use in areas such as Southeast Asia, which are experiencing continued avian transmission and occasional spread to humans.

A live, attenuated vaccine would more likely produce antibodies after 1 injection and would have a number of other theoretical advantages over inactivated vaccine in a pandemic. Unfortunately, such a vaccine will not generally be considered for 2 reasons. First, production requires specific pathogen–free eggs and these will be in shorter supply than ordinary eggs. This could change if cell culture could be used. However, the bigger problem involves evaluation before and use early in the pandemic. Since this vaccine virus could reassort, it might introduce the pandemic virus into the population if used too early. The question also arises whether attenuation would be successful with a new and potentially more virulent wild type, a result which could be evaluated in advance in animals (*38*).

## Antiviral Drugs in the Pandemic

While supplies of vaccines will increase as the pandemic evolves, antiviral drug supplies will decrease as stockpiles are depleted. The starting level will depend on the amount of stockpiling, based more on economic and policy consideration than science. As with vaccines, planning decisions will be in place to prioritize use during the initial period, which may need to be modified based on epidemiologic characteristics of the outbreak and clinical characteristics of the cases. The key virologic issue will be whether the pandemic strain is susceptible to the antiviral drugs. Most recent planning, since it is focused on the threat of the A (H5N1) virus, has assumed that adamantanes would not be useful. This assumption means that if the disease is systemic and case fatality is high, among the NAIs only oseltamivir would be useful, since it is absorbed (*39*). Given the limited quantities likely to be available, at least in the near future, the drug will have to be restricted to treat those most likely to die or have severe consequences. Careful observation of treatment results will help to determine if the dose and duration of therapy is appropriate. Seasonal prophylaxis uses larger quantities of drug, but possibly limited postexposure use could be feasible. Zanamivir, if available, might find its role in prevention. Infection is likely through the respiratory tract, and given past evidence, the drug could make a major contribution in prophylaxis before vaccine is available. Throughout, mechanisms need to be in place to monitor antiviral resistance, which might emerge as a problem with extensive use of the drugs. A long-term goal should be to develop new antiviral agents against influenza. The global reliance on basically 1 drug from 1 source cannot be allowed to continue. Other NAIs are available for clinical evaluation, and drugs targeting other phases of influenza viral replication would be especially useful.

Given the threat of a virulent virus such as A (H5N1) and the suggestion that adaptation to transmissibility may occur gradually, the concept has emerged that antiviral drugs may be used to interrupt early, local transmission. The aim would be to prevent spread out of the region of origin, in other words, extinguishing the epidemic at its source (*40*). Transmission models suggest that this strategy will work as long as the *R*_o_ or basic reproductive number is not high (*41*). Thus, this goal seems worthy of consideration on more than a theoretical basis. Models also suggest that the approach might be more likely to succeed with partial immunity in the population (*42*). This immunity could be produced by prior vaccination with a current A (H5N1) vaccine. Practical issues may be of greatest concern, especially the ability to put antiviral prophylaxis in place rapidly in rings around cases. Supplies of oseltamivir are also an issue. Will those countries with stockpiles be willing to share with other countries on the possibility, not certainty, that a pandemic could be avoided?

## Conclusion

Major challenges are presented in controlling a pandemic with vaccine and antiviral drugs, particularly one caused by an A (H5N1) virus similar to those currently circulating. Some are specific to the particular intervention, but others are more generic. Long-term needs exist, such as developing innovative technologies for vaccine prevention and designing antiviral drugs to affect different targets. However, immediate attention for vaccines must be directed to a coordinated international approach to vaccine evaluation, paying attention to ways in which the least amount of virus can immunize the largest number of persons. Use of a possibly unmatched A (H5N1) vaccine for priming should be considered, especially in Southeast Asia, or other areas with the most pressing need. In those regions, antiviral strategies need to be evaluated; drug studies in animal models will be necessary, given the sporadic nature of the disease in humans. Overall, developing countries will have limited access to vaccines and antiviral drugs, and their needs must not be forgotten. With marginal healthcare infrastructures, they will suffer the most, whatever the severity of the pandemic.
